# Revisiting Electronic Topological Transitions in the Silver–Palladium (Ag_*c*_Pd_1−*c*_) Solid Solution: An Experimental and Theoretical Investigation

**DOI:** 10.3390/ma17112743

**Published:** 2024-06-04

**Authors:** Florian Reiter, Alberto Marmodoro, Andrei Ionut Mardare, Cezarina Cela Mardare, Achim Walter Hassel, Arthur Ernst, Martin Hoffmann

**Affiliations:** 1 Institute of Chemical Technologies of Inorganic Materials (TIM), Johannes Kepler University (JKU), Altenberger Straße 69, 4040 Linz, Austria; andrei.mardare@jku.at (A.I.M.);; 2 Institute of Physics (FZU) of the Czech Academy of Sciences, Cukrovarnická 10, 16253 Prague, Czech Republic; 3 New Technologies Research Centre, University of West Bohemia, CZ-301 00 Pilsen, Czech Republic; 4 Czech Technical University, Trojanova 339, 120 00 Praha, Czech Republic; 5 Faculty of Medicine and Dentistry, Danube Private University (DPU), Steiner Landstraße 124, 3500 Krems an der Donau, Austria; 6 Institute for Theoretical Physics, Johannes Kepler University (JKU), Altenberger Straße 69, 4040 Linz, Austria; 7 Max-Planck-Institut für Mikrostrukturphysik, Weinberg 2, 06120 Halle, Germany

**Keywords:** Ag-Pd alloy, DFT calculation, concentration, electronic topological transitions (ETT)

## Abstract

Multiple thick film samples of the ${\mathrm{Ag}}_{c}{\mathrm{Pd}}_{1-c}$ solid solution were prepared using physical vapour deposition over a borosilicate glass substrate. This synthesis technique allows continuous variation in stoichiometry, while the distribution of silver or palladium atoms retains the arrangement into an on-average periodic lattice with smoothly varying unit cell parameters. The alloy concentration and geometry were measured over a set of sample points, respectively, via energy-dispersive X-ray spectroscopy and via X-ray diffraction. These results are compared with ab initio total energy and electronic structure calculations based on density functional theory, and using the coherent potential approximation for an effective medium description of disorder. The theoretically acquired lattice parameters appear in qualitative agreement with the measured trends. The numerical study of the Fermi surface also shows a variation in its topological features, which follow the change in silver concentration. These were related to the electrical resistivity of the ${\mathrm{Ag}}_{c}{\mathrm{Pd}}_{1-c}$ alloy. The theoretically obtained variation exhibits a significant correlation with nonlinear changes in the resistivity as a function of composition. This combined experimental and theoretical study suggests the possibility of using resistivity measurements along concentration gradients as a way to gain some microscopic insight into the electronic structure of an alloy.

## 1. Introduction

Silver (Ag) and palladium (Pd) are both noble metals with a relatively high electrochemical potential, i.e., they are not readily reacting with oxygen or water. Silver is used as a unique material with its selectivity to ethylene in the selective partial oxidation to yield ethylene oxide [1]. Other applications rely on the oligodynamic effect in which very small amounts of silver ions are released that have a strong impact on microorganisms. This is why thin silver fibers in cotton are used to improve the wound healing in medical applications; they can also hinder bacteria from digesting sweat in socks resulting in olfactoric challenges. Palladium also has a lot of applications as a catalyst. While homogeneous catalytic use, e.g., for cross-coupling, is important in synthetic chemistry, it is, in particular, its use in heterogeneous catalysts such as the three-way catalytic converter, the Lindlar catalyst, that makes this element so important. It shows a unique affinity to hydrogen and acts as a hydrogen-permeable membrane at higher temperatures. This effect can be used to purify hydrogen-containing gases. The combination of these two properties make Pd the element of choice for hydration and dehydration reactions.

While the importance of each of the elements alone is already impressive, it must be emphasized that the possibility to form binary alloys over a wide range of compositions allows the properties to be tuned to the actual needs. Silver reacts easily with H_2_S and forms highly insoluble AgS as a black coverage. This tarnishing can be suppressed when alloying Ag with Pd [2].

The electronic structure of Ag-Pd catalysts and electrocatalysts is highly important as they are used for a large range of reactions, namely improved ethylene epoxidation [3], reductive homocoupling of alkyl bromides [4], selective reductive homocoupling of alkyl iodides [5], electrocatalytic de-/hydrogenation [6] or dichlorination of dichlorophenoxyacetic acid [7], the direct synthesis of hydrogen peroxide [8] and ultrastable ${\mathrm{CO}}_{2}$ reduction to formate [9].

Besides the direct metallurgical alloying, Ag-Pd alloys can also be prepared through electroless plating [10], e.g., against biofouling [11], or through DC sputtering, e.g., as a lubricant in high-temperature applications [12]. Thin films of Ag-Pd can be used to improve the SERS activity [13] or to prepare flexible electrically conductive fibers [14].

The affinity to hydrogen can be used not only in the above-mentioned catalytic reaction but also for hydrogen sensing [15]. On the other hand, the adsorption can already change the Ag-Pd thin film surface through segregation depending on the composition [16]. Ag-Pd films are hydrogen permeable but the permeability depends on the alloy’s composition and structure [17], and the structure may change upon loading and discharging [18]. Theoretical work supports the experimental findings that a non-ideal absorption effect occurs at temperatures below 300 °C [19]. Experimental support can be found from NMR measurements, which showed more than one possible jump process [20]. Of high practical interest is the fact that the three hydrogen isotopes H, D and T show different permeation rates [21], which is considered for the treatment of water containing tritium, e.g., from the Fukushima nuclear power plant [22]. Given the importance of Ag-Pd membranes for applications at higher temperatures, a number of investigations have been performed to show how Ag-Pd alloys behave upon heating [23] and their thermal stability [24].

Variations in the composition and stoichiometry of metallic alloys allow the optimization of materials’ properties for a variety of applications. The experimental study of impurities, defects and material properties in disordered systems and their theoretical description is a fundamental part of material science. At a microscopic level, these features are mainly determined by the electronic structure. The density of states (DOS), the electronic band structure and the shape and topology of the Fermi surface provide, in particular, very useful parameters to understand how certain macroscopic features emerge, and can potentially be tuned in the desired directions.

Variations in the composition and stoichiometry of metallic alloys allow to optimize materials properties for a variety of applications. The experimental study of impurities, defects and material properties in disordered systems and their theoretical description are a fundamental part of material science. At a microscopic level, these features are mainly determined by the electronic structure. The density of states (DOS), the electronic band structure and the shape and topology of the Fermi surface provide in particular very useful parameters to understand how certain macroscopic features emerge, and can potentially be tuned in the desired directions.

Previous studies [25] have introduced the idea that electronic topological transitions (ETTs), i.e., the variations in the shape and connectivity of the Fermi surface, play an important role in controlling the bulk modulus and other mechanical properties of a metal. ETTs can be caused by doping of pure metals. In addition, transport properties such as the electrical resistivity undergo significant changes. While applied pressure and in situ/operando spectroscopy can provide ways to, respectively, sweep through and characterize these ETTs within a single sample, the tuning of stoichiometry offers a permanent route to the engineering of the desired features in an alloy, or conversely, to infer from simple conductivity measurements other features of interest which also originate from the same microscopic physics.

An ideal scenario for the cross-validation of this type of investigation is offered by metallic solid solutions such as ${\mathrm{Ag}}_{c}{\mathrm{Pd}}_{1-c}$, where the constituents remain highly miscible over a continuum of concentration values. Earlier numerical studies [26,27,28,29] highlighted the connection between the topology of the Fermi surface and the properties of an alloy, such as the bulk modulus. Following on from those studies, we propose here a systematic comparison between experiments and ab initio theoretical descriptions, which aims to carefully validate the connection between subtle features of the alloy’s electronic structure and its measured lattice parameters and the literature values of the resistivity.

In our study, we combine experiments and theory in the following way. First, ${\mathrm{Ag}}_{c}{\mathrm{Pd}}_{1-c}$ alloys were fabricated using vapor deposition, and then the structure and stoichiometry of the ${\mathrm{Ag}}_{c}{\mathrm{Pd}}_{1-c}$ alloys were studied with energy-dispersive X-ray spectroscopy and with X-ray diffraction. The obtained results were compared with first-principles calculations for total energy and structure. Further, the electronic structure and transport properties were studied from first principles and compared with experiments reported in early studies.

Various samples of the ${\mathrm{Ag}}_{c}{\mathrm{Pd}}_{1-c}$ solid solution have been prepared by means of physical vapor deposition (PVD) [30] over a borosilicate glass substrate in order to obtain a continuous lateral concentration gradient between pure silver and pure palladium. The resulting alloy thin films have been characterized by measuring local thickness and the corresponding stoichiometry and crystalline lattice parameters, respectively, through energy-dispersive X-ray spectroscopy (EDX) and through X-ray diffraction (XRD). On the numerical side, we adopted the effective medium description offered by the coherent potential approximation (CPA) [31] in combination with density functional theory (DFT), implemented within a self-consistent Korringa–Kohn–Rostoker (KKR) Green’s function framework.

Comparing experimentally and theoretically obtained structural properties, we conclude that our first-principles approach can adequately describe the ground state properties of ${\mathrm{Ag}}_{c}{\mathrm{Pd}}_{1-c}$ alloys. Further electronic and transport properties of the ${\mathrm{Ag}}_{c}{\mathrm{Pd}}_{1-c}$ alloys were studied from first principles and the results were compared with previous experiments.

This combined study allows the experimental values of the resistivity to be related to ETTs that manifest as changes in the shape and connectivity of the electronic Bloch spectral function (BSF) at the Fermi level [32]. In particular, we improve our earlier study of these effects [29] by taking into account here the relativistic effects by numerically solving the Kohn–Sham problem in terms of the Dirac equation [33]. Previous ab initio results based on the scalar relativistic (SR) approximation [34] could show the relationship between some ETT features and changes in the calculated bulk modulus. Here, it is shown how the fully relativistic (FR) approach produces a more accurate and realistic band structure in comparison with experimental results. The importance of fully relativistic effects appears, in particular, in the Pd-rich region of the Ag-Pd solid solution, where FR calculations of the Fermi surface show, for instance, a neck around the L point of the Brillouin zone which is not visible at the SR level. Furthermore, FR calculations allow the degeneracy in the electronic pocket around the X point to be resolved (see Figure 2 in Ref. [28]).

This paper is structured as follows. The experimental results are first presented in Section 2. The outcomes of SR and FR ab initio calculations, highlighting in particular the role of ETTs as a function of Ag concentration, are then examined in Section 3. Section 4, at the end, proposes a summary of the main findings and our conclusions from the study.

## 2. Samples’ Preparation and Characterization

PVD in the form of thermal co-evaporation was performed over a borosilicate glass substrate in order to prepare thick films of ${\mathrm{Ag}}_{c}{\mathrm{Pd}}_{1-c}$ with a continuous change in concentration along one dimension of the glass strip, which serves as a substrate [30]. This sample preparation technique allows us to maintain at all intermediate concentrations the reference fcc unit cell structure common for both pure Ag or pure Pd. Alternative growth methods, such as sputtering or even electron-beam vaporization, would instead result in amorphous alloy phases [35] unsuitable for the present studies. Three distinct samples were produced and characterized, each spanning an interval of Ag concentrations chosen in partial overlap with the other ones in order to allow cross-validation of the results. In each case, the film thickness, its stoichiometry and its fcc lattice parameter were measured at multiple points, i.e., over a range of concentrations between pure Pd and pure Ag extremes. Each aspect of this procedure is outlined in further detail in the following.

### 2.1. Sample Growth via Physical Vapour Co-Deposition (PVD)

**Figure 1:**
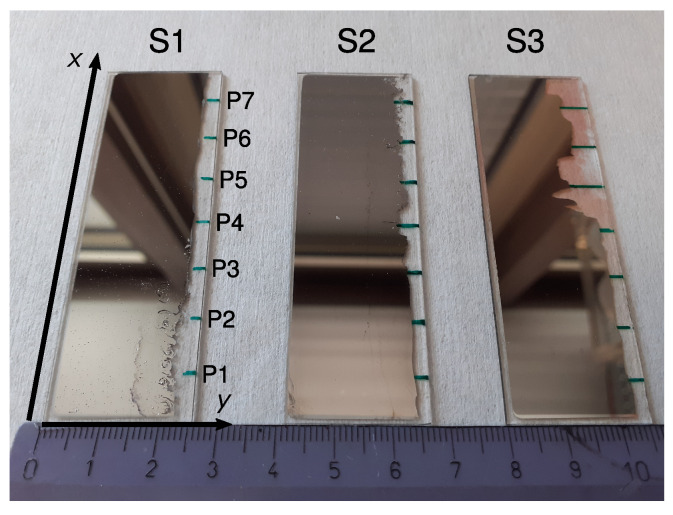
Photograph of samples S1, S2 and S3. On the blank edges (right), the measurement points (labeled P1–P7 in S1) were marked as green lines on the backside of the glass plates. A ruler with marks every centimeter is shown at the bottom for scale. Reddish regions in sample S3 stem from silver salts caused by a reaction with the glass plate. These do not affect further measurements however.

Ag and Pd were deposited on top of three microscope slides of borosilicate glass with dimensions 76 mm × 26 mm × 1 mm. These are referred to as the samples S1, S2 and S3 in the following. This growth results in a smoothly varying atomic concentration between 0 and 1 along the *x* direction, aiming for an intermediate Ag concentration (middle point in Figure 1) of about 10 at.% for sample S1, 50 at.% for sample S2 and 90 at.% for sample S3. The end and start points of S1, S2 and S3 were prepared with some overlap in concentration along *x* for cross-validation purposes across samples.

The deposition was performed by placing pure metallic Ag and Pd in a boron nitride crucible and by thermally evaporating inside the Thermal Co-Evaporation Chamber (THEO) within the CALMAR cluster [36]. The deposition rates of Ag and Pd are listed in Table 1, together with the initial and final pressures during the de position. The completed Ag-Pd libraries are shown in Figure 1.

The samples S1 and S2 exhibit defects in the form of shrinkholes. They occur with a higher density at the surface in regions with a high Pd concentration. This is a consequence of the higher melting point of palladium and is in agreement with Grüneisen’s rule [37]. When the temperature is lowered, Pd tends to solidify before Ag. This leads to the contraction of its occupied volume, resulting in local distortions of the lattice and, in particular, the formation of holes at the surface.

After the evaporation, an EDX (Section 2.3) was performed to map the concentration along the samples. The Ag-Pd layer was then partially removed as shown on the right edges of the samples in Figure 1 and seven spots P1–P7 at 1 cm intervals along the *x* side (green marks in Figure 1) were chosen for the characterization steps detailed in Section 2.2 and Section 2.4 below.

### 2.2. Film Thickness

We assessed whether the samples can be considered as bulk-like by first measuring the thickness of each ${\mathrm{Ag}}_{c}{\mathrm{Pd}}_{1-c}$ film using contact profilometry at the measurement points P1–P7 along the *x* side of the samples (Table 2).

**Figure 2:**
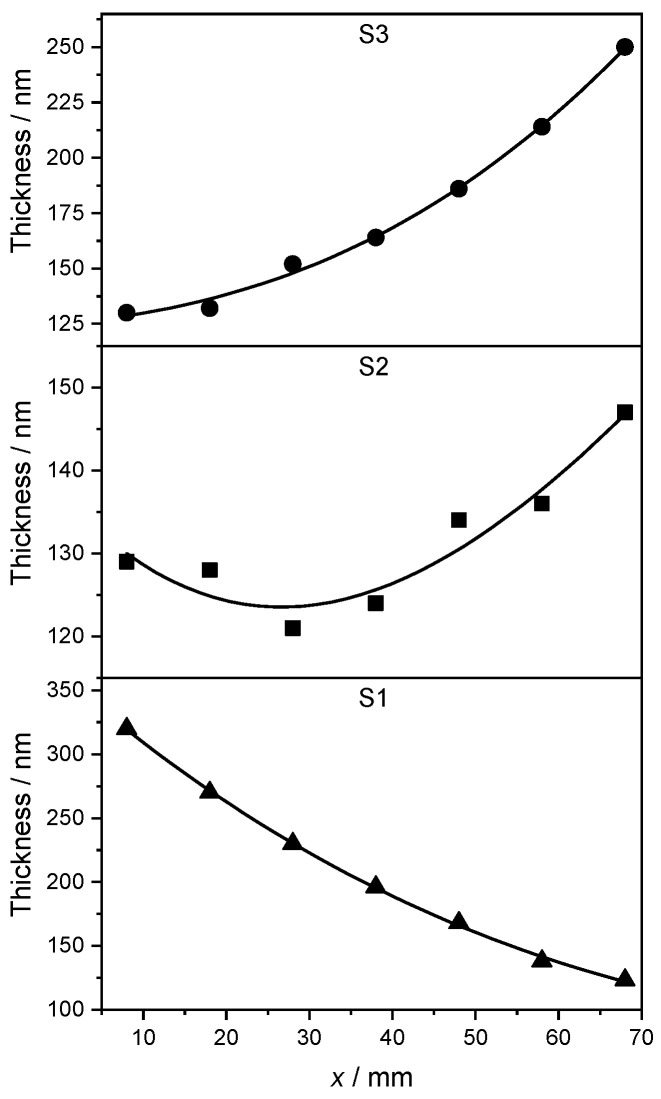
Thickness of the Ag-Pd films as a function of position along the *x* direction. At *x* = 8 cm (measurement point P1) in S1 and at *x* = 68 cm (measurement point P7) in S3, the composition was, respectively, pure Pd and almost pure Ag, see Table 3. The concentration in S2 along the *x* direction varies according to Table 3, too. A cubic B-spline is shown as continuous black line to show the trend.

The larger differences in the Ag-Pd layer thickness for the samples S1 and S3 are due to the geometry of the PVD setup and to the different deposition rates required to achieve the desired stoichiometry (see Section 2.1). The variable thickness along the *x* side of each strip is plotted in Figure 2. A cubic B-spline is shown as a continuous black line along with the data to highlight the trend. Overall, the thickness of the AgPd films is confirmed to be larger than 120 nm throughout all samples. This corresponds to approximately 300 atomic layers, allowing a bulk-like description of the material to be adopted in the theoretical study of Section 3.

### 2.3. Stoichiometry via Energy-Dispersive X-ray Diffraction (EDX)

**Figure 3:**
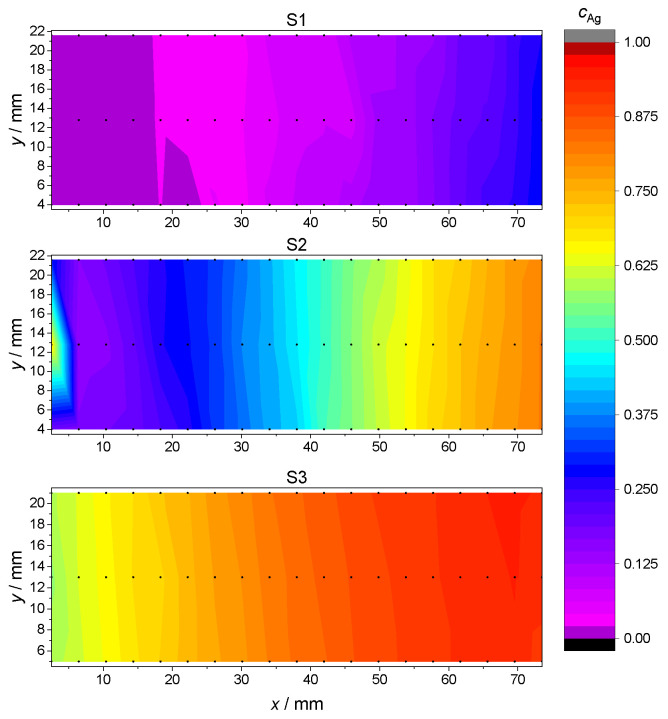
Ag concentration for each sample S1, S2 and S3 as a function of (x,y) location at the glass plate. Actual 19×3 EDX measurements points are marked in black. Intermediate values are interpolated using the Renka–Cline method [38].

The Ag concentration of each sample was determined as a function of position along the strip by means of EDX mapping [36]. This technique was favored over more surface sensitive methods, like Auger electron spectroscopy (AES), since the composition at the surface is likely to not be representative of the interior of the samples. The EDX spectrum was acquired at 19×3 measurement points along the *x* and *y* dimensions of each sample, selecting, in particular, the Lα and Lβ transitions of Ag and Pd around E≈3 keV. In addition to these dominant peaks, a signal from O, K, Na, Si and Ca was also observed and interpreted as being due to the composition of the borosilicate glass substrate. The atomic concentration of Ag at each measurement point can be obtained from the corresponding EDX spectrum by the formula

(1) $$c_{\mathrm{Ag}}=\frac{n_{\mathrm{Ag}}}{n_{\mathrm{Ag}}+n_{\mathrm{Pd}}},$$

where $n_{\mathrm{Ag}}$ and $n_{\mathrm{Pd}}$ denote the relative amount of Ag and Pd atoms, respectively. The concentration gradients for the samples S1, S2 and S3 are plotted in Figure 3, using the Renka–Cline method for results’ interpolation between discrete measurement points [38].

This measurement confirmed that the S1, S2 and S3 samples spanned, respectively, an atomic concentration of Ag in the range from 0 to ≈0.25, from 0.20 to 0.75 and from 0.65 to 0.95, with a fairly constant Ag concentration along the shorter *y* dimension. In the discussion of subsequent measurements and data analysis, the Ag concentrations depending on the measurement points P1–P7 are the arithmetic average of the interpolated values along the *y* side for each point P1–P7. The so-obtained Ag concentration values as a function of measurement positions P1–P7 are reported in Table 3.

In order to highlight the quality of the concentration gradient within each sample, the measured Ag concentrations in the middle of the *y* direction (see measurement points in Figure 3) are fitted. Due to the geometry of the PVD setup, the concentration behavior along the *x* direction is expected to follow a $\sin^{2}$ trend [36] given by

(2) $$c_{\mathrm{Ag}}\left(x\right)=c_{0}+A\cdot \sin^{2}\left(\frac{\pi \cdot (x-x_{c})}{w}\right).$$

**Figure 4:**
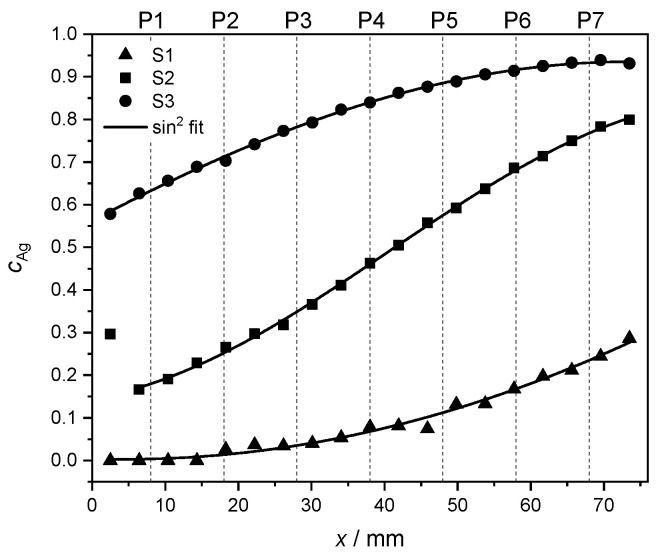
Interpolated Ag concentrations from EDX measurements (see Figure 3) at the *y* center of each of the samples S1 (▲), S2 (■) and S3 (●), including a fit to Equation 2 (continuous black line, see text). The dashed lines mark the positions P1–P7 at which the film thickness and the fcc lattice parameter were measured.

The outcome of the numerical fit is shown as a continuous black line on top of the measured data in Figure 4. In Equation (2), the parameter $c_{0}$ stands for a concentration offset at $x=x_{c}$. The fitting parameter $x_{c}$ accounts for the position where the $\sin^{2}$ curve reaches a minimum. The parameter *A* denotes the concentration range for each set of values. Finally, the parameter *w* is proportional to the FWHM of the $\sin^{2}$ curve and thus serves as a measure of width.

For sample S1, the larger uncertainties in the fit can be explained by the narrower range of concentrations in comparison to the other samples, and thus, to relatively bigger fluctuations in the concentration values. The first measurement point of S2 was rejected for the fit to Equation (2) due to its unusually high Ag concentration, also visible in Figure 3. The corresponding data were, however, retained in other characterization and data analysis steps. The individual fitting parameters together with their uncertainties are reported in Table 4.

All samples appear to follow a concentration trend in good agreement with Equation (2). Since the subsequent XRD characterization is sensitive to the composition of each strip along its whole *y* side, the estimated stoichiometry at points P1–P7 is given in Table 3 by taking an average of the corresponding interpolated EDX values as described earlier.

### 2.4. Lattice Parameter via X-ray Diffraction (XRD)

**Figure 5:**
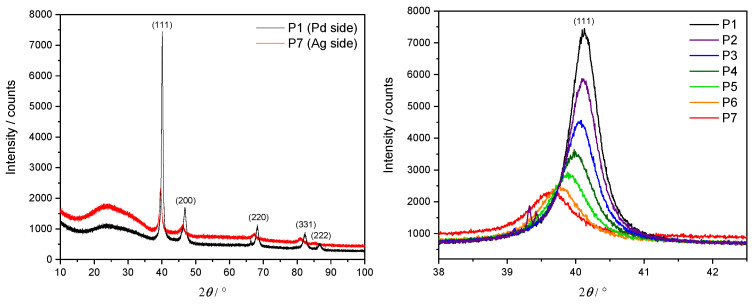
XRD diffractogram of S1. **Left panel**: full angular sweep at the P1 (black) and P7 (red) measurement points. **Right panel**: detailed results around the main peak at 2θ≈40°, corresponding to reflection at Miller indices (h,k,l) = (1, 1, 1).

In order to confirm an fcc lattice structure and investigate the relationship between its lattice parameter and the Ag concentration, an XRD was performed at each measurement point P1–P7 along the three samples. Here, a Cu $K^{\alpha }$ X-ray source (λ=0.1541874 nm) with Bragg–Brentano geometry was utilized. The lateral resolution of the X-ray beam of approximately 5 mm motivated the choice of spacing steps at 1 cm intervals along the *x* direction. Example results in the form of an XRD diffractogram from S1 are depicted in Figure 5. The left panel shows the diffraction intensity over a complete angular sweep for the two extremal points P1 (pure Pd, in black) and P7 ($c_{\mathrm{Ag}}^{\left(1\right)}=20.791$ at.%, in red).

A clearly dominant peak appears at 2θ≈40°, corresponding to the Miller indices (h,k,l)=(1,1,1) for an fcc unit cell. Other smaller peaks at diffraction angles of 2θ≈48°, 68°, 82° and 88° are also visible and can be interpreted as originating from Ag and Pd for the Miller indices (200), (220), (311) and (222), respectively. A significantly wider and weaker peak round 2θ≈25° is a consequence of the borosilicate glass substrate. This analysis confirms the persistence of the fcc lattice geometry as the Ag concentration increases. A comparison of the P1 dataset with powder diffraction files (PDFs) for pure Pd [39] supports the assessment of having sufficiently thick films for bulk-like interpretation of the given results.

The right panel focuses on the main peak at 2θ≈40°, showing in particular its gradual drift towards smaller angles for higher concentrations of Ag.

The fcc lattice parameter for each diffractogram is calculated by evaluating the Bragg condition on the position of the main peak

(3) $$2d\sin(\theta_{\max})=n\lambda ,$$

where *d* is the distance between atomic layers in the direction of out-of-plane, $\theta_{\max}$ is the angle of maximum intensity and *n* is the order of diffraction. Solving Equation (3) for *d*, under the assumption of n=1 and an fcc unit cell, yields the equation for the lattice parameter *a*:

(4) $$a=d\cdot \sqrt{h+k+l}=\frac{\lambda \sqrt{3}}{2\sin(\theta_{\max})}.$$

**Figure 6:**
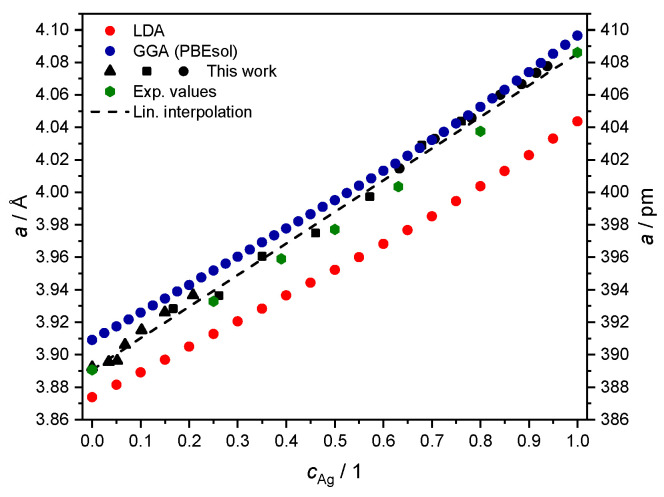
Theoretical values of the fcc lattice parameter *a*, from fitting the equation of state with total energies computed with either LDA (red) or GGA-PBEsol (blue) adopted as exchange-correlation functional. Experimental values from Section 2.4 in black (▲ for S1, ■ for S2 and ● for S3) and from Ref. [40] (green hexagons) are plotted for comparison.

Resulting values for the fcc lattice parameter as a function of Ag concentration are given in Table 5, and plotted together with literature data from Ref. [40] for cross-comparison in Figure 6.

In this representation, the deviation in the literature values (green hexagons) from the simplified linear trend of Vegard’s law [41] is visible as a convexity in the estimated lattice parameter, always lying below the straight line between the $c_{\mathrm{Ag}}=0$ and $c_{\mathrm{Ag}}=1$ limits [40,42,43]. The data from the XRD measurement are comparable in magnitude but do not exhibit the expected deviation from Vegard’s law. We speculate that this may be due to contractive stress, also deemed responsible for the macroscopic shrinkhole defects visible in samples S1 and S2 (see Figure 1). Although further investigations may be needed to clarify this aspect of the problem in detail, we consider the agreement with other measurements of the fcc lattice parameter for the ${\mathrm{Ag}}_{c}{\mathrm{Pd}}_{1-c}$ system good enough to be taken as input for the next numerical part of our study in Section 3.

## 3. Results from Ab Initio Calculations and Comparison

We calculated the ground state properties of the ${\mathrm{Ag}}_{c}{\mathrm{Pd}}_{1-c}$ solid solution using DFT within the multiple scattering/Green’s function formulation [44]. Within this framework, different exchange-correlation functionals are made available by the LIBXC package [45] and we discuss the results from two possible choices in Section 3.1.

In a similar fashion, we compared the results for calculations of the electronic Bloch’s spectral function when using a scalar relativistic (SR) [34] and a fully relativistic (FR) [46] formulation of the Kohn–Sham problem.

The FR treatment adopts the relativistic Green’s function corresponding to the solution of the Dirac–Kohn–Sham Hamiltonian, rather than the spin-polarized SR Schrödinger Hamiltonian.

In all cases, we resorted to the coherent potential approximation (CPA) [47,48] in order to describe any intermediate value of *c* in the ${\mathrm{Ag}}_{c}{\mathrm{Pd}}_{1-c}$ alloy without the discretization and the computational burdens posed by supercells of matching stoichiometry. Specific numerical parameters of particular relevance in controlling the quality of self-consistency are reported in the corresponding section below.

### 3.1. Total Energy Calculations and Estimate of Equilibrium fcc Lattice Parameter vs. Alloy Concentration

For each concentration value $c_{\mathrm{Ag}}$ the DFT total energy was minimized over a set of 25 fixed values of the fcc lattice parameter *a*, assigned by linear interpolation $a_{\mathrm{lin}}=c_{\mathrm{Ag}}a_{\mathrm{Ag}}+\left(1-c_{\mathrm{Ag}}\right)a_{\mathrm{Pd}}$ of the literature values for pure Ag and pure Pd [42]. This part of the work resorted to the full-charge density approximation (FCDA) [49] for more realistic results compared to the muffin tin or atomic sphere approximations as discussed in, e.g., Ref. [29].

The quality of convergence was verified as a function, in particular, of sampling resolution in performing Brillouin zone integration for the valence charge density, and truncation in angular momentum for the Green’s function and multiple scattering expansion in spherical harmonics. Sufficient numerical quality appears to have been reached by using a sampling mesh of $80^{3}$ *k*-points over the whole Brillouin zone, and by representing the problem up to $l_{\max}=3$ over the spherical harmonics basis set of the multiple scattering expansion.

We report here the results produced with either the local density approximation [50] (LDA) or the PBEsol version of the generalized gradient approximation [51] (GGA) as exchange-correlation functionals.

The corresponding set of 25 values for total energies vs. the lattice parameter was used as input for the numerical fit of the Birch–Murnaghan equation of state [52] to determine, in particular, the unit cell equilibrium volume and the bulk modulus (not discussed here), as detailed in Ref. [29]. This procedure to estimate the fcc lattice parameter *a* was repeated over 21 values of the Ag concentration (at steps of 5 at.%) for LDA calculations, and over 41 values (at denser steps of 2.5 at.%) for GGA (PBEsol) calculations. The outcome is plotted together with experimental data from Section 2.4 in Figure 6.

The procedure for the ${\mathrm{Ag}}_{c}{\mathrm{Pd}}_{1-c}$ alloy yields the usual outcome of LDA, underestimating the equilibrium lattice parameter, as discussed in better detail in, e.g., Ref. [53]. The slope in dependence on Ag concentration is instead fairly similar, and it also reproduces the outcome of measurements from Section 2.4 and from the literature values from Ref. [40] (Figure 6). Both choices of the exchange-correlation functional lead to a larger deviation from the experimental data for high concentrations of Ag. This feature might be a consequence of how valence electrons are treated as non-interacting quasi-particles in DFT [54]. As revealed by XPS measurements of bulk Ag [55], this transition metal has a fairly localized DOS at the Fermi level, mainly associated with 4d electrons. This makes it more challenging to handle correlation effects through the above local or semi-local DFT functionals [56], compared to the case of bulk Pd.

However, the calculated crystalline structure as a function of concentration is in a very good agreement with the current experiment. Therefore, we conclude that our computational approach can adequately describe the ground state properties of ${\mathrm{Ag}}_{c}{\mathrm{Pd}}_{1-c}$ alloys. Next, we study further the electronic structure and transport properties using the PBEsol density functional in the full relativistic approach.

### 3.2. Electronic Topological Transitions via the BSF

We performed electronic BSF calculations using the effective ${\mathrm{Ag}}_{c}{\mathrm{Pd}}_{1-c}$ potentials produced with the more realistic PBEsol exchange-correlation functional, and adopting the associated theoretical fcc lattice parameter discussed in Section 3.1 (see Figure 6).

**Figure 7:**
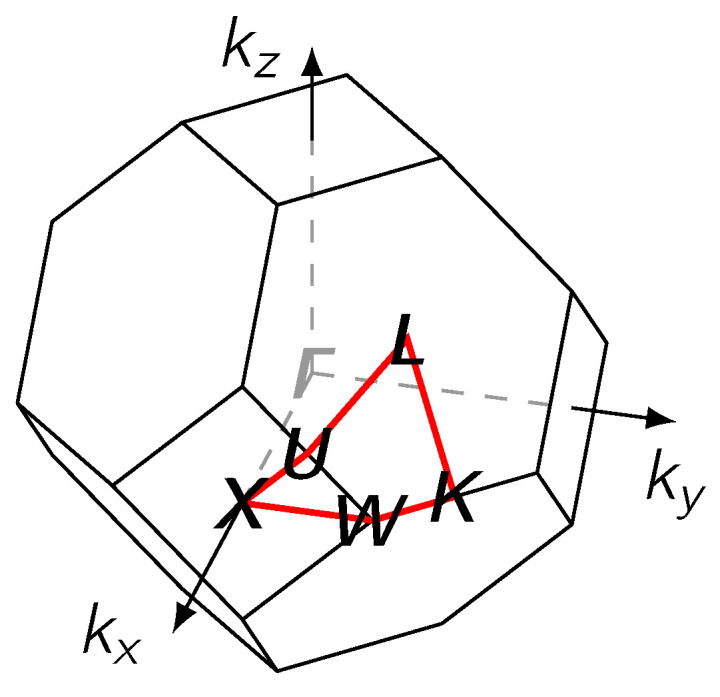
The first Brillouin zone for an fcc lattice with the path K−L−U−X−K along high symmetry points on the surface (red).

The electronic structure of the alloy was examined at the Fermi level $E_{F}$, adopting the same closed path on the surface of the first Brillouin zone (Figure 7) and other representation conventions as in Ref. [29]. Along the horizontal axis we show how results change over 41 values of the concentration, sampling the range from pure Pd to pure Ag at steps of 2.5 at.%. The resulting plot shows in color the appearance or disappearance of intensity peaks for the BSF, as it is recomputed along the chosen path across high-symmetry points (vertical axis).

In particular, we identify the occurrence of an ETT at a certain concentration when either two BSF maxima merge or one BSF maximum splits as a function of varying Ag concentration. These results are compared upon employing either the SR (similar to Ref. [29]) or the FR treatment, in which one solves the Dirac Green’s function for electrons [46].

### 3.3. Relation to Resistivity

**Figure 8:**
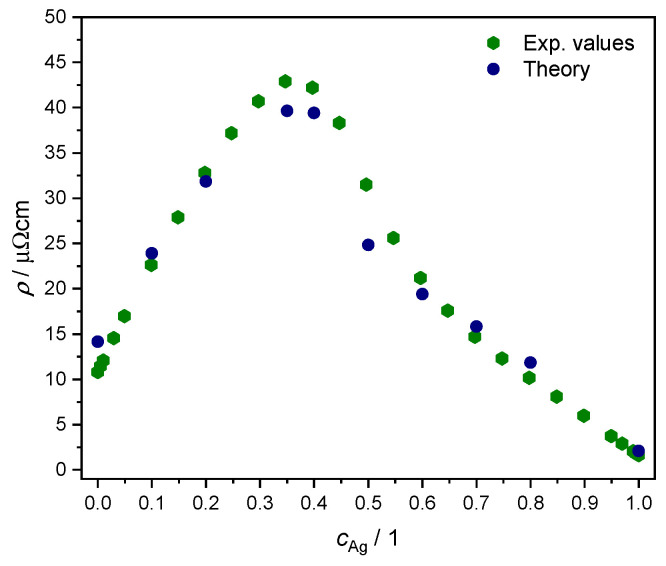
Comparison of ${\mathrm{Ag}}_{c}{\mathrm{Pd}}_{1-c}$ resistivity as a function of Ag concentration, between experimental values from Ref. [60] (green hexagons) and our own ab initio results from linear response theory for T=300 K (blue circles); see text.

We adopted the linear response formalism of Ref. [57], inclusive in particular of disorder vertex corrections as implemented in the SPRKKR electronic structure code [44,58], to calculate the resistivity as a function of concentration for T=300 *K*. Due to the symmetries of the fcc lattice, the resulting 3×3 conductivity tensor in x, y, z is non-zero only along the diagonal [59], with alike values within numerical accuracy. The results are plotted in Figure 8 together with experimental values from Ref. [60]. We note the overall quantitative agreement, confirming the quality of the KKR-CPA effective medium description in representing the disordered AgPd alloy.

**Figure 9:**
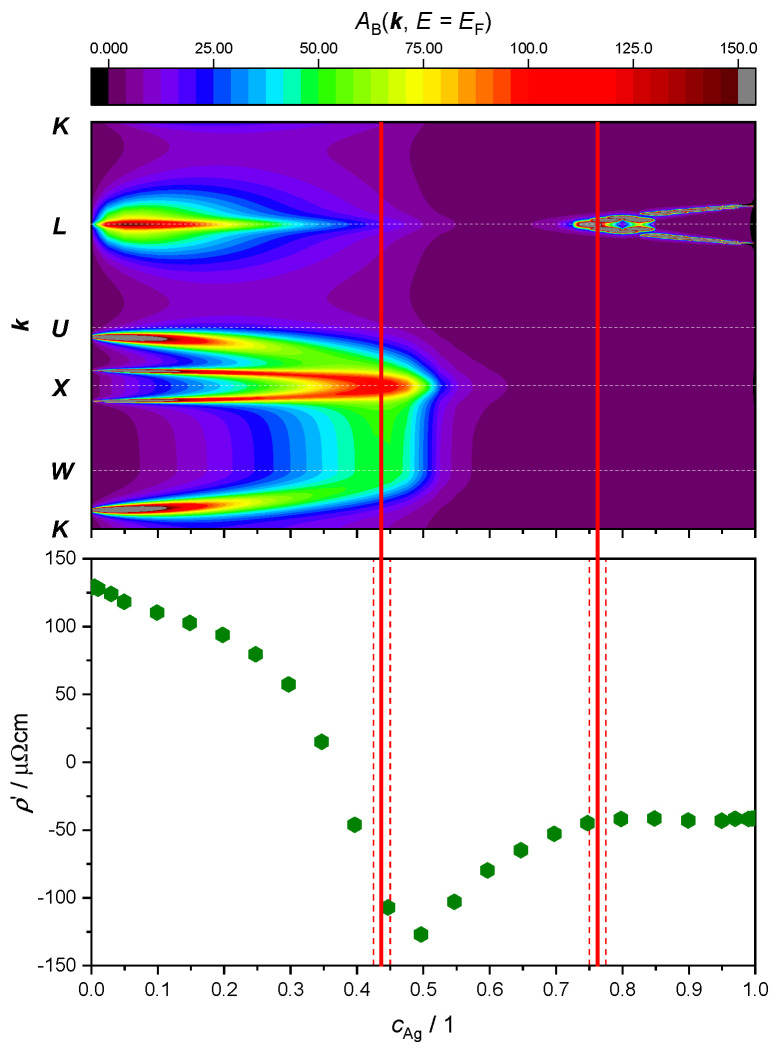
**Top panel**: intensity of the electronic BSF (color coded, in arbitrary units) along the K−W−X−U−L−K path (vertical axis, see Figure 7) calculated in the SR approximation and plotted against Ag concentration (horizontal axis). **Bottom panel**: derivative of the experimental resistivity from Ref. [60]. Concentration intervals in which peaks have merged or split are marked by red dashed lines, with ETT estimated to occur in the middle (continuous red line).

**Figure 10:**
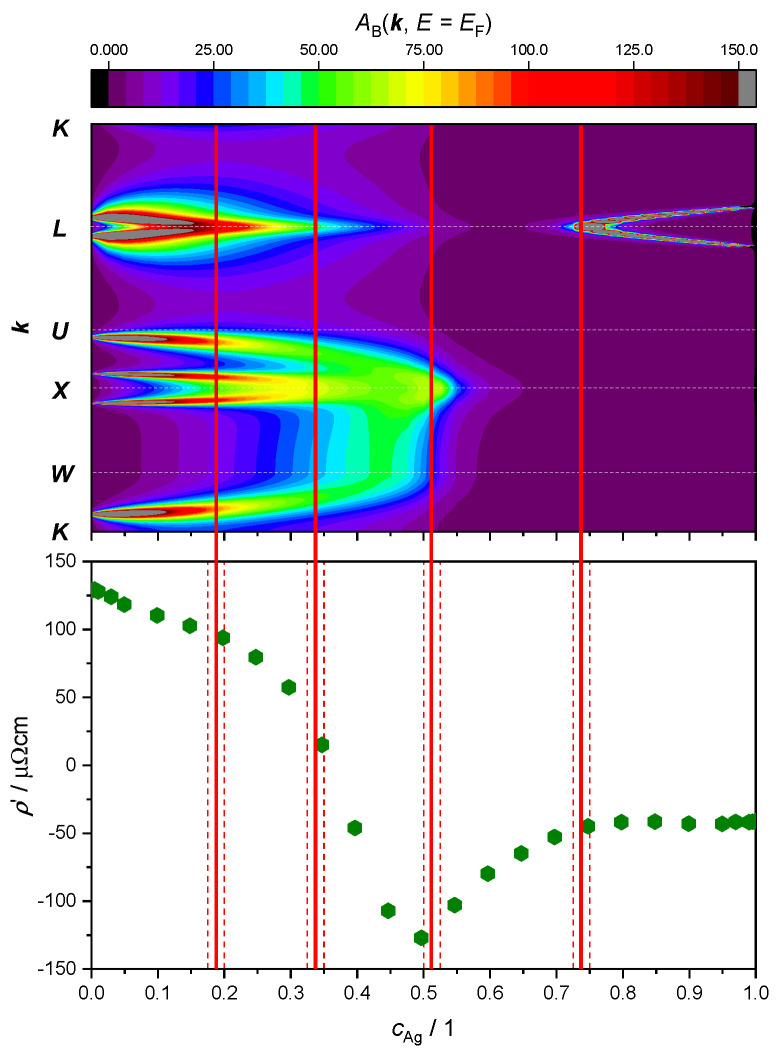
**Top panel**: intensity of the electronic BSF (color coded, in arbitrary units) along the K−W−X−U−L−K path (vertical axis, see Figure 7) calculated in the FR approximation and plotted against Ag concentration (horizontal axis). **Bottom panel**: same data as in Figure 9. Concentration intervals in which peaks have merged or split are marked by red dashed lines, with ETT estimated to occur in the middle (continuous red line).

Here, our focus lies in studying the relationship between ETTs and the nonlinear changes in the resistivity of the AgPd alloy, as a function of Ag concentration. This is illustrated by plotting both the evolution of the BSF peaks at $E_{F}$ and the derivative of the $\rho^{\prime }=\frac{d}{{\mathrm{dc}}_{\mathrm{Ag}}}\rho$ as a function of Ag concentration (Figure 9 and Figure 10). The upper panel provides theoretical criteria to identify Ag concentration values for which ETTs occur. These are marked by vertical red lines placed in the middle of the concentration interval (red dashed lines) in which the corresponding ETT was found. The bottom panel highlights the possible concomitant variation in the measured electrical transport properties (here: resistivity) of the material.

**Figure 11:**
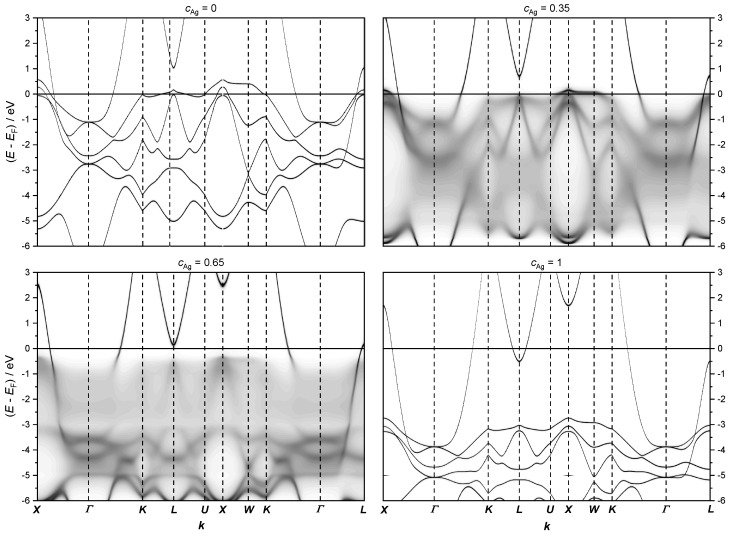
The fully relativistic electronic structure of the AgPd alloy calculated along the path ***X*** − ***Γ*** − ***K*** − ***L*** − ***U*** − ***X*** − ***W*** − ***K*** − ***Γ*** − ***L*** on the first Brillouin zone (see Figure 7) for the concentrations $c_{\mathrm{Ag}}$ = 0, 0.35, 0.65 and 1, respectively.

The fully relativistic electronic structure of AgPd alloys, presented in Figure 11, indicates that the most important changes in the Fermi level happen along the K−L−U−X−W−K path of the Brillouin zone.

Note that the band structure below the Fermi level for $c_{\mathrm{Ag}}$ = 0.35 and 0.65 appear blurred due to CPA.

At low Ag concentrations, the energy dispersion close to the Fermi level is mainly given by flat 4 *d* bands of Pd, which do not contribute much to the conductivity due to the low velocity of these states. Therefore, if these states are located at the Fermi level, the resistivity increases. According to our calculations, the flat Pd 4 *d* states cross the Fermi level in the range of Ag concentration between $c_{\mathrm{Ag}}=$ 0.3 and $c_{\mathrm{Ag}}=$ 0.5. To analyze the most important changes in the electronic structure at the Fermi level, the calculations were refined only along a path across the high-symmetry points K−L−U−X−W−K for various concentrations of Ag (see Figure 9 and Figure 10).

It is noted that a quantitative estimate of transport properties by means of a mean free path treatment would involve an actual Brillouin zone volume integral.

We perform this comparison resorting to both the SR (as conducted in Ref. [29]) and the FR approximation for BSF calculations, using GGA (PBEsol) for the exchange-correlation functional and adopting the associated theoretical fcc lattice parameters from Section 3.1 (see Figure 6).

The SR treatment adopted in Figure 9 leads to the identification of only two topological transitions. The first one occurs between $c_{\mathrm{Ag}}=$ 42.5 at.% and $c_{\mathrm{Ag}}=$ 45.0 at.%, where a double peak around the X-point merges as the amount of Ag increases. The second ETT occurs between 75.0 at.% and 77.5 at.%, where a new peak appears and then splits into two sharp peaks around the L-point.

Looking at the derivative of the resistivity, we notice a minimum at $c_{\mathrm{Ag}}\approx$ 50 at.%, which could be associated with the first ETT. Another change in this slope around $c_{\mathrm{Ag}}\approx$ 75 at.% appears to correspond with the second ETT. No other ETTs are predicted by the SR approximation for the BSF. In particular, the kink in the $\rho^{\prime }$ curve around $c_{\mathrm{Ag}}\approx$ 25 at.%, as well as the maximum in the resistivity curve (i.e., $\frac{\rho }{c_{\mathrm{Ag}}}=$ 0) around 35 at.%, would remain unexplained.

Repeating the BSF calculations with the FR treatment (Figure 10) leads to the identification of two additional ETTs. Starting again from the $c_{\mathrm{Ag}}=$ 0, i.e., the pure Pd limit, the small-radius Fermi surface neck around the L-point is now visible (see also Figure 2 in Ref. [28]). This feature shrinks with increasing Ag concentration and finally disappears between $c_{\mathrm{Ag}}=$ 17.5 at.% and $c_{\mathrm{Ag}}=$ 20.0 at.%. This change in electronic structure takes place at the same Ag concentration where the first slope change in the $\rho^{\prime }$ curve was noticed, around 25 at.%. The second additional ETT resolved by the FR treatment occurs between $c_{\mathrm{Ag}}=$ 32.5 at.% and $c_{\mathrm{Ag}}=$ 35.0 at.%. The two peaks around the X point merge in a similar fashion as noted at $c_{\mathrm{Ag}}\approx$ 45 at.% in the SR outcome of Figure 9. The difference to the SR case is that in the FR picture, the double peak around the X point merges before the broad peaks around U and W move towards the X point. The resistivity of the alloy has its maximum at a similar value of the concentration $c_{\mathrm{Ag}}\approx$ 35 at.% (Figure 8), where the derivative passes through the zero. Considering the neighborhood of the X point, with increasing Ag concentration the broad peaks coming from U and W move towards X, producing again a double peak around the point X. The forming of this double peak O=is completed before the Ag concentration has reached 50 at.% and merges into a single peak between 50 at.% and 52.5 at.%. This ETT can be associated with the minimum of the $\rho^{\prime }$ curve. An ETT in this concentration region was already visible in the SR outcome of Figure 9 (top panel), but it appears more spread-out in the FR treatment. The subtle change appears to correlate better with the minimum of $\rho^{\prime }$ at $c_{\mathrm{Ag}}\approx$ 50 at.%. The fourth and final ETT occurs with the merging of the peaks around the L point between $c_{\mathrm{Ag}}=$ 72.5 at.% and $c_{\mathrm{Ag}}=$ 75.0% and is associated with the kink in the $\rho^{\prime }$ curve at $c_{\mathrm{Ag}}\approx$ 75 at.%.

An explanation of the mechanism relating changes in the alloy resistivity as a function of Ag concentration with the appearance of ETTs might be based on a mean free path model of electric transport [61]. This approach estimates the conductivity to be proportional to the product between the number of charge carriers at the Fermi level, their Fermi velocity and the inverse of the Bloch’s relaxation time due to disorder. All such parameters can, in principle, be quantitatively extracted from the BSF, and systematically compared with more accurate linear response calculations for the study of alloys as a function of composition [62].

The results from Figure 10 (upper panel) graphically suggest a decrease in the number of states at the Fermi level when the Ag concentration increases until the first ETT is produced around the L point between $c_{\mathrm{Ag}}=$ 17.5 at.% and 20 at.%. Subsequently, spectral weight is mainly located around the U−X−W region of the Brillouin zone, but it reaches much lower intensity and it shows large smearing as a function of disorder. Together, these two effects produce the resistivity maximum around $c_{\mathrm{Ag}}=$ 35 at.%. For higher Ag concentrations, the intensity remains approximately constant with a simultaneously increasing number of valence electrons. This suggests a higher number of charge carriers near the Fermi level, which is associated with a decrease in the resistivity with a constant slope up to $c_{\mathrm{Ag}}\approx$ 50 at.%, where another ETT occurs. The merging of the double peak causes fewer possible states at the Fermi level and causes the increasing slope of the resistivity. Finally, the slope of the resistivity remains roughly constant, i.e., the conductivity increases in linear fashion beyond $c_{\mathrm{Ag}}=$ 75 at.%. Since the intensity of the BSF remains approximately constant, this behavior can again be explained by a higher number of electrons at the Fermi level with increasing Ag concentration.

## 4. Conclusions

We have prepared, experimentally characterized and studied from first principles the prototypical solid solution alloy ${\mathrm{Ag}}_{c}{\mathrm{Pd}}_{1-c}$ over the whole range of concentrations between the pure Pd and pure Ag limits.

Measurements of EDX and XRD confirm, respectively, that the thick film synthesis via PVD has achieved the desired continuous variation in stoichiometry and that samples have retained the fcc lattice structure for all compositions.

Ab initio calculations of total energies via DFT, and subsequent fitting of the Birch–Murnaghan equation of state, reproduce the experimental trend, in further agreement with the pre-existing literature [29,60].

We cross-compare this outcome upon using two different exchange-correlation functionals, LDA and GGA, and find the usual result of a better description of equilibrium lattice parameters with the second choice.

A good agreement of computed structural properties with the current experiment validates our first principles approach for treatment of the ${\mathrm{Ag}}_{c}{\mathrm{Pd}}_{1-c}$ alloys. From this, further electronic and transport properties were calculated with the proposed density functional approach within the coherent-potential approximation.

We then considered the electric resistivity of the alloy, validating the quality of the CPA effective medium description by comparing the measurements with linear response calculations inclusive of disorder vertex corrections [57].

Finally, we examined the electronic structure of the alloy in terms of its BSF at the Fermi level, and studied, in particular, how ETTs appear as a function of concentration and seem to correlate with changes in the slope for the resistivity. The results comparing an SR and an FR treatment of the Kohn–Sham problem reveal a richer picture in the latter case, which follows more closely the above experimental changes in conductivity trends.

## Figures and Tables

**Table 1:** Deposition rates of Ag ($R_{\mathrm{Ag}}$) and Pd ($R_{\mathrm{Pd}}$) for the samples S1, S2 and S3 used during PVD. The base pressure was $5\cdot 10^{-6}\;\mathrm{hPa}$ and did not increase above $6\cdot 10^{-6}\;\mathrm{hPa}$ during the deposition.

Sample	$R_{\mathrm{Ag}}$/nm $\cdot s^{-1}$	$R_{\mathrm{Pd}}$/nm$\cdot s^{-1}$
S1	0.114	0.89
S2	0.43	0.37
S3	0.87	0.084

**Table 2:** Thickness $d_{i}$ of the ${\mathrm{Ag}}_{c}{\mathrm{Pd}}_{1-c}$ film for the samples S1, S2 and S3 measured at the points P1–P7 (see Figure 1).

Point	*x*/mm	$d_{1}$/nm	$d_{2}$/nm	$d_{3}$/nm
P1 (Pd side)	8	320	129	130
P2	18	270	128	132
P3	28	230	121	152
P4	38	196	124	164
P5	48	168	134	186
P6	58	138	136	214
P7 (Ag side)	68	123	147	250

**Table 3:** Atomic Ag concentration $c_{\mathrm{Ag}}^{\left(i\right)}$ for the samples i=1,2,3 at each measurement point P1–P7. Reported values are an average along the *y* side of the strips, see text.

Point Nr.	$c_{\mathrm{Ag}}^{\left(1\right)}$/at.%	$c_{\mathrm{Ag}}^{\left(2\right)}$/at.%	$c_{\mathrm{Ag}}^{\left(3\right)}$/at.%
1 (Pd side)	0	16.704	63.358
2	3.404	26.158	70.567
3	5.165	35.031	78.189
4	6.759	46.055	84.148
5	10.182	57.235	88.517
6	14.944	67.947	91.548
7 (Ag side)	20.791	76.117	93.805

**Table 4:** Numerical fitting parameters including uncertainties and quality of the fit to Equation (2) for the data in Figure 4.

Parameter	S1	S2	S3
$c_{0}$/1	0.003±0.007	0.133±0.016	−0.4±0.8
*A*/1	247±132,409	0.72±0.03	1.4±0.8
$x_{c}$/mm	4±6	−8.0±2.9	−140±80
*w*/mm	$6500\pm 1.7\cdot 10^{6}$	194±11	420±150
red. $\chi^{2}$	$1.19\cdot 10^{-4}$	$4.25\cdot 10^{-5}$	$2.34\cdot 10^{-5}$

**Table 5:** The diffraction angle of maximum intensity $\theta_{\max}$ and the corresponding fcc lattice parameter calculated via Equation (4) from XRD data acquired at the positions P1–P7 in all three samples S1, S2 and S3, see text.

	S1	S1	S2	S2	S3	S3
Position	$2\theta_{\max}$/°	a/Å	$2\theta_{\max}$/°	a/Å	$2\theta_{\max}$/°	a/Å
P1 (Pd side)	40.127	3.89231	39.743	3.92838	38.855	4.01457
P2	40.091	3.89566	39.659	3.93636	38.671	4.03294
P3	40.083	3.89641	39.407	3.96053	38.543	4.04582
P4	39.979	3.90613	39.259	3.97486	38.403	4.06001
P5	39.883	3.91515	39.031	3.99717	38.339	4.06653
P6	39.767	3.92610	38.711	4.02893	38.271	4.07349
P7 (Ag side)	39.655	3.93674	38.563	4.04380	38.231	4.07759

## Data Availability

Data are contained within the article.
